# First Report on the Molecular Detection and Characterization of *Rickettsia felis* in Laelapidae (Acari: Mesostigmata) Mites in Malaysia

**DOI:** 10.3390/vetsci12050443

**Published:** 2025-05-06

**Authors:** Hiryahafira Mohamad Tahir, Faraliana Che Lah Ernieenor, Suhaili Zainal Abidin, Vishalani Vishnu Narainasamy, Mariana Ahamad

**Affiliations:** Acarology Unit, Infectious Diseases Research Centre, Institute for Medical Research (IMR), National Institutes of Health, Ministry of Health Malaysia, Shah Alam 40170, Selangor, Malaysia; erniee@moh.gov.my (F.C.L.E.); suhaili.za@moh.gov.my (S.Z.A.); vishalani@moh.gov.my (V.V.N.); marianaa@moh.gov.my (M.A.)

**Keywords:** *Laelaps*, *Rickettsia felis*, small mammals, vector-borne diseases

## Abstract

Rickettsiae are Gram-negative and obligate intracellular bacteria that cause rickettsioses. These pathogens are typically transmitted by arthropod vectors, such as ticks, mesostigmatid mites, and fleas. Rickettsiae are responsible for many emerging infectious diseases worldwide and are the second most frequently reported cause of non-malarial febrile illnesses in Southeast Asia. However, in Malaysia, studies on the prevalence and distribution of rickettsiae have primarily focused on humans, with limited data on these bacteria in vectors and small mammal hosts. Thus, this study aims to investigate the presence of *Rickettsia* spp. in small mammals and their associated mesostigmatid mites collected from potential rickettsioses areas in Selangor. Animal trapping was conducted across three different ecological study sites comprising a recreational area, agricultural land, and coastal area. A total of 41 small mammals and 363 mesostigmatid mites were collected and identified. Ten percent of the total individual mites were processed for morphological examination, and the remaining mites were then pooled by hosts, with five individual mites per tube, for DNA extraction. The collected samples, comprising blood, animal tissue, and pooled mites, were subjected to DNA extraction and were screened for *Rickettsia* spp. via nested polymerase chain reaction (PCR), targeting the citrate synthase-encoding gene (*gltA*) and outer membrane protein B gene (*ompB*). Interestingly, two pools (3.33%) of *Laelaps* spp. recovered from *Maxomys whiteheadi* and *Bandicota indica*, collected from a recreational area, tested positive for *Rickettsia* spp. Sequence analysis and phylogenetic tree of the *ompB* gene revealed the presence of *Rickettsia felis* in both laelapid mite pools. To our knowledge, this study provides the first molecular detection of *R. felis* in *Laelaps* spp. in Malaysia.

## Simple Summary

This study investigates the presence of *Rickettsia* bacteria, which cause diseases transmitted by arthropods, such as ticks, mites, and fleas. These bacteria are a significant cause of illness worldwide and are especially common in Southeast Asia. However, in Malaysia, most research has focused on human infections, with little information about how these bacteria spread through animals and insects. The results showed that two pools of mites from the recreational area tested positive for *Rickettsia felis*, a type of *Rickettsia* bacteria. This finding is the first detection of *R. felis* in mesostigmata mites in Malaysia. This research is important because it helps us understand how bacteria spread in animals and their vectors, providing valuable information for controlling these diseases and protecting public health.

## 1. Introduction

*Rickettsia* is a genus of obligate intracellular Gram-negative bacteria that are present worldwide, except in Antarctica [1,2,3]. These bacteria are primarily known as human pathogens vectored mainly by hematophagous arthropods, such as ticks, fleas, and mites, causing significant public health problems due to rickettsial diseases worldwide [4,5,6]. Notably, in Southeast Asia, rickettsial diseases rank as the second most frequently reported infection among non-malarial febrile illnesses [7].

Previously, the genus *Rickettsia* was conventionally categorised into three groups according to their phenotypic traits: the spotted fever group (SFG), the typhus group (TG), and the scrub typhus group (STG). However, recent advances in genomic analyses have led to the reorganisation of the taxonomy of rickettsiae into four distinct phylogenetic groups: (i) SFG, primarily linked with ticks but occasionally with fleas and mites; (ii) the TG, encompassing the causative agents of epidemic typhus and murine typhus associated with lice and fleas; (iii) an ancestral group containing *Rickettsia bellii* and *Rickettsia canadensis*; and (iv) a transitional group represented by *Rickettsia akari* and *Rickettsia felis* [8]. The STG was proposed as a novel genus named *Orientia* [9].

*Rickettsia felis* is one of the rickettsiae that belong to the TG group, which is principally associated with *Ctenocephalides felis*, also known as the cat flea [10]. This pathogen is widely regarded as an emerging human pathogen, with a rickettsial disease referred to as flea-borne spotted fever, cat flea typhus, and *R. felis* rickettsiosis [11,12]. Although *C. felis* is currently known as the primary vector and reservoir host of *R. felis*, this rickettsial DNA has been detected in ticks and mites, suggesting a wide range of arthropod hosts [8,13].

Mesostigmatid mites (Acari: Mesostigmata) are small arachnids (average body length of 0.5 mm) that are abundant and diverse, encompassing a wide range of lifestyles and habitats worldwide [14,15]. Most species within this group are predatory, with a significant portion also acting as parasites or symbionts of mammals, birds, reptiles, and other arthropods [16]. Mites of the family Laelapidae are distributed worldwide, with species like *Laelaps echidninus* and *Laelaps nuttalli* being hematophagous parasites on humans and rodents [17]. The role of mesostigmatid mites in the circulation of some disease agents has been previously confirmed, and recent studies have also demonstrated that mesostigmatid mites may be reservoirs as well as vectors of some pathogenic rickettsiae [8]. Although various rickettsiae have recently been detected in Laelaps mites, investigations on this topic remain very scarce, particularly in Southeast Asian.

In Malaysia, investigations into the prevalence and distribution of *Rickettsia* spp. have focused predominantly on humans and have shown a high prevalence of rickettsioses among indigenous communities in rural areas [18,19,20]. However, limited information is available on the animal reservoirs and vectors of this rickettsial species in acari, especially mesostigmatid mites. To date, no *Rickettsia* spp. have been detected in mesostigmatid mites in Malaysia, probably due to few screenings of mites for rickettsioses compared to ticks and fleas. Thus, the present study aimed to investigate the presence of *Rickettsia* spp. in mesostigmatid mites and their small mammals collected from Selangor.

## 2. Materials and Methods

### 2.1. Study Area

**Figure 1:**
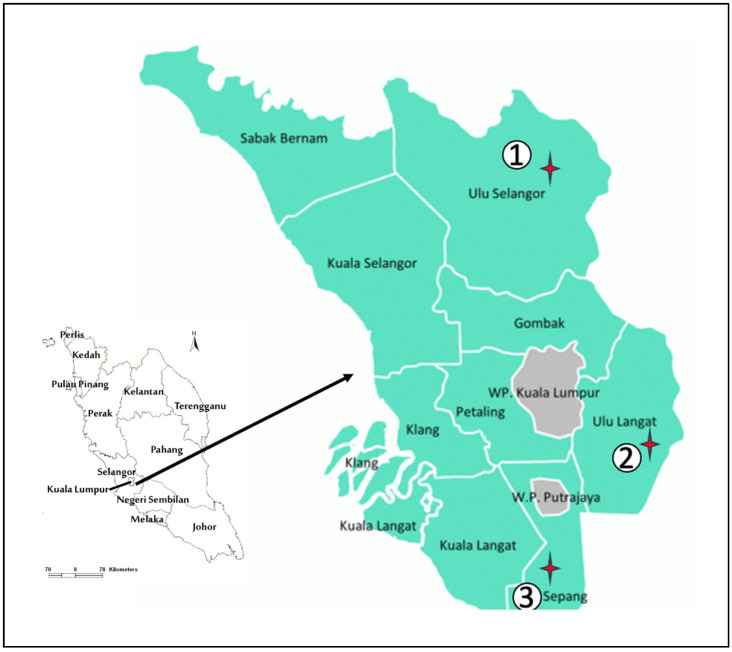
Maps showing the three localities (labelled with a red star) of small rodent trapping in Selangor: (1) Sungai Kedondong-Ulu Selangor district (3.4300° N, 101.7301° E), (2) Beranang -Ulu Langat district (2.8780° N, 101.8699° E), and (3) Bagan Lalang-Sepang district (2.6059° N, 101.6983° E).

Selangor is a state located on the west coast of Peninsular Malaysia, with a diverse ecology. In this study, animal trapping was conducted between October 2024 and December 2024 at three distinct ecological sites: Sungai Kedondong (SK), Beranang (BR), and Bagan Lalang (BL), representing a recreational area, agricultural land, and coastal area, respectively (Figure 1). The selection of study sites was based on criteria such as potential areas with high risks of rickettsial infection where humans and rodents coexist, and ecosystems with high acari infestations, as documented in previous sampling by the Acarology Unit, Institute for Medical Research (IMR).

### 2.2. Trapping of Small Mammals and Collection of Mites

All procedures involving animals were conducted and followed under the Animal Regulations & Ethical Consideration, as suggested by the Animal Care & Use Committee (ACUC). A total of 50 wire traps (20 cm × 20 cm × 30 cm) were set up at each study site, which used ripe bananas as bait to attract small mammals. The traps were checked once daily in the morning for four consecutive trapping days. All trapped small mammals were placed in white cloth bags and returned to the Acarology Unit. The animals were anaesthetized with diethyl ether before the mesostigmatid mites were screened via a stereomicroscope (Model Stemi DV4 Zeiss, Jena, Germany). The small mammals were thoroughly examined for mesostigmatid mites by brushing off from hosts using the comb and direct observation of their bodies. The mites were subsequently collected using sterile soft forceps or sharpened wooden applicator sticks and placed into 1.5 mL tubes containing 70% ethanol. Each tube was labelled with a specific number corresponding to the animal. Following examination, blood, kidney, spleen, and liver tissues of small mammals were collected and stored at −20 °C for subsequent pathogen screening. The species of animals were identified by morphological traits following [21,22,23].

### 2.3. Morphological Identification of Mesostigmatid Mites

The total number of mesostigmatid mites recovered from each animal host was recorded. A 10% sub-sample of adult-stage mesostigmatid mites was randomly selected and mounted on a glass slide, while the remaining mites (300 mites) were then pooled by host with five individual mites per tube (60 tubes) for DNA extraction prior to *Rickettsia* detection. The mites were cleaned in a cavity block containing lactophenol, and the lateral margins were punctured with an applicator stick to facilitate the penetration of the cleaning agents [24]. The mites were left in lactophenol for two to three days to ensure complete clearing of their bodies. For mounting, a drop of Hoyer’s medium was placed on a clean glass slide, and one of the ventral views of the mites was transferred. A coverslip was then placed over the medium. The slides were examined under a high-resolution microscope (Nikon ECLIPSE Ni-U microscope, Tokyo, Japan) at 40× magnification. The slide-mounted mesostigmatid mites were identified to the genus level based on their external morphological characteristics using taxonomic keys [25].

### 2.4. DNA Extraction of Mesostigmatid Mites

All DNA extraction from the mesostigmatid mites, blood, and animal tissue was performed according to the manufacturer’s protocol using QIAGEN Dneasy Blood & Tissue Kit (Qiagen, Dusseldorf, Germany). Briefly, five adult-stage mesostigmatid mites collected from the same individual animal host were pooled in a 1.5 mL Eppendorf tube (300 mites = 60 pools). The specimens were washed in 70% ethanol and rinsed with distilled water five times. Mesostigmatid mites were then macerated in 45 μL of ATL buffer using a sterile micro pestle under a microscope. The lysates were incubated with Proteinase K at 56 °C for 3 h, followed by nucleic acid purification according to the manufacturer’s instructions. The DNA was eluted in 50 μL of elution buffer and then stored at −20 °C until further use as a PCR template.

### 2.5. DNA Extraction from Blood

The total genomic DNA was extracted from 200 μL of blood mixed with 200 μL of AL buffer and 20 μL of proteinase K in a microcentrifuge tube. The mixture was vortexed for 15 s prior to incubation at 56 °C for 10 min. Further DNA extraction was performed according to the Qiagen kit extraction protocols recommended by the manufacturer. The extracted DNA samples were eluted in 200 μL of elution buffer and then stored at −20 °C until further use as a template for PCR amplification.

### 2.6. DNA Extraction from Animal Tissue

DNA was extracted from the kidney, liver, and spleen of each animal by weighing approximately 500 mg of each tissue and homogenizing them in PBS buffer. Then, the subsequent DNA extraction was performed following the same protocol used for mesostigmatid mites.

### 2.7. Detection of Rickettsia spp. Via Nested PCR

Nested PCR was performed to detect *Rickettsia* spp. in mesostigmatid mites, blood, and tissue (kidney, liver, and spleen) of small mammals. The presence of *Rickettsia* spp. DNA in all samples was screened using the citrate synthase gene (*gltA*), common to the *Rickettsia* genus, and the outer membrane protein gene (*ompB*), common to all SFG and TG rickettsiae. Nested PCR was conducted following [26], in which RpCS.877p and RpCS.1258n were used as outer primers and RpCS.896 and RpCS.1233n were used as inner primers in the *gltA* gene. For the *ompB* gene, the outer primers were rompB OF and rompB OR, while the inner primers were rompB SFG IF and rompB SFG/TG. To prevent cross-contamination, DNA extraction, PCR master mix preparation, thermal cycling, and PCR product observation were performed in separate rooms. The first PCR reactions were conducted in a total volume of 25 μL containing 12.5 μL 2× Dream Taq Mix (Thermoscientific, Waltham, MA, USA), 1 μL of each 10 μM primer, 5.5 μL nuclease-free water, and 5 μL of DNA as a template. One-tenth of the total volume from the first PCR products was used as a DNA template for the second PCR. Synthetic DNA (gBlock) of the partial *ompB* gene of *Rickettsia* spp. (Accession No. AF123724.1) and sterile distilled water were used as positive and negative controls in each PCR reaction, respectively. The PCR reactions for both cycles were as follows: initial denaturation at 95 °C for 5 min, followed by 35 cycles of denaturation at 95 °C for 15 s, annealing at 54 °C for 15 s, and extension at 72 °C for 30 s, with a final extension at 72 °C for 3 min after completion of all cycles. The PCR was conducted using a Master Cycler Personal machine (Eppendorf, Hamburg, Germany). The PCR products were visualized in 1.5% agarose gel electrophoresis, stained with SYBR^®^ Safe DNA gel stain (Invitrogen, Waltham, MA, USA), and viewed by the Gel Doc Imaging System (Analytik Jena, Jena, Germany).

### 2.8. DNA Sequencing and Phylogenetic Analysis

All positive PCR amplicons with the expected size corresponding to *Rickettsia* species were then sent to Apical Scientific, Malaysia, for DNA sequencing. The sequencing chromatograms were then analyzed using Chromas Lite software version 2.1 and exported as FASTA sequence files. The obtained sequences were then compared with GenBank entries using the Basic Local Alignment Search Tool (BLAST) provided by the National Centre for Biotechnology Information (NCBI) (https://blast.ncbi.nlm.nih.gov/Blast.cgi) (URL accessed on 2 February 2025) for the confirmation and identification of pathogens. Later, the phylogenetic tree was constructed using the neighbour-joining (NJ) method via Molecular Evolutionary Genetics Analysis (MEGA) software to determine the genetic relationships between positive *Rickettsia* species and reference strains. The NJ bootstrap values were estimated using 1000 replicates with Kimura’s two-parameter model substitution (K2P distance).

### 2.9. Data Analyses

The total number of mesostigmatid mites was counted for each small mammal captured. The infestation rate of small mammals with mesostigmatid mites was calculated according to [27] using the formula below:

$$\mathrm{Infestation}\;\mathrm{rate}=\frac{Number\;of\;small\;mammals\;infested\;with\;mites}{Total\;number\;of\;small\;mammals\;captured}\times \mathrm{100}$$

The positivity rates of mites and animal hosts infected with *Rickettsia* spp. were calculated according to [28] using the following formula:

$$\mathrm{Positivity}\;\mathrm{rate}=\frac{Number\;of\;positive\;sample}{Total\;number\;of\;samples\;examined}\times \mathrm{100}$$

### 2.10. Statistical Analysis

Fisher’s Exact Test was performed using IBM SPSS Statistics Version 29.0 for Windows (IBM Corp., Armonk, NY, USA) to assess the association between localities and infestation rates. A *p*-value of less than 0.05 was considered statistically significant, indicating a significant association between infestation rates and localities.

## 3. Results

### 3.1. Infestation Rate of Small Mammals with Mesostigmatid Mites

A total of 41 small mammals were collected at three localities in Selangor, of which nine were infested with mesostigmatid mites, resulting in an overall infestation rate of 21.96% (Table 1). Sungai Kedondong (SK) had the highest infestation rate of 12.20%, followed by Beranang (BR) and Bagan Lalang (BL), both with an equal infestation rate of 4.88%. The relationship between localities and infestation rate was determined using Fisher’s Exact Test, and there was a significant association between the localities and the infestation rate (*p* = 0.002) (Supplementary Materials Figure S1).

Throughout the sampling, four species of rodents belonging to the family Muridae comprising *Rattus rattus*, *Rattus tiomanicus*, *Bandicota indica*, *Maxomys whiteheadi*, and one species of tree shrew, *Tupaia glis*, from the family Tupaiidae, were examined for mesostigmatid mites. The most abundant small mammal species collected was *R. tiomanicus*, followed by *T. glis*, *R. rattus*, *M. whiteheadi*, and *B. indica*. Among the rodents, *M. whiteheadi* showed the highest infestation rate (7.32%), followed by *B. indica*, *R. tiomanicus*, and *R. rattus* with a similar percentage (4.88%). Interestingly, no mite infestation was detected in 13 individuals of *T. glis*. A total of 363 mesostigmatid mites were collected, with the highest number of mites found in *B. indica* (n = 201), followed by *M. whiteheadi* (n = 126), *R. tiomanicus* (n = 26), and *R. rattus* (n = 10).

### 3.2. Morphological Characterization

**Figure 2:**
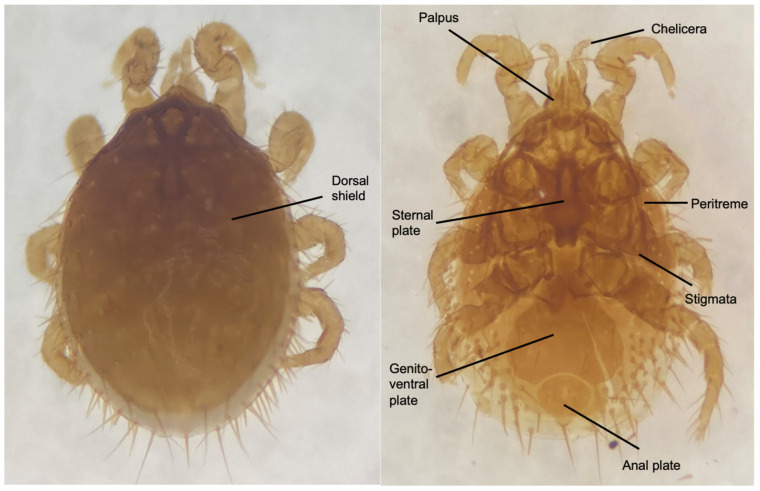
External morphological characteristics of adult female *Laelaps* sp. on dorsal (**left**) and ventral (**right**) views.

In this study, only one genus of *Laelaps* sp. was identified based on its morphological characteristics using specific taxonomic keys. *Laelaps* sp. have a rounded body shape and prominent dorsal setae (Figure 2). The gnathosoma of *Laelaps* sp. carries feeding appendages known as chelicerae and palp. On the dorsal view, both males and females have a single dorsal shield (thickened chitinous area) that protects from desiccation and predation. The idiosome of adult female *Laelaps* sp. can be divided into three parts: the sternal plate, genito-ventral plate, and anal plate. The sternal plate is located anteriorly and is typically larger, bearing distinct setae or spines, which may vary in size or shape depending on the species, followed by the genito-ventral plate, which connects to the genital area. The anal plate of *Laelaps* spp. is located at the posterior end of the idiosoma and may have a few short setae, and its shape can vary, with setae along its margins. The spiracular openings, known as stigmata, function in gaseous exchange and serve as a major diagnostic feature for distinguishing between the orders of acari. In the order mesostigmata, the stigmata are positioned between coxae III–IV and are associated with an elongated anteriorly directed groove known as the peritreme.

### 3.3. Prevalence of Rickettsia spp. in Mesostigmatid Mites and Small Mammals

There was no *Rickettsia* DNA detected in 41 tissues (spleen, kidney, and liver) and 36 blood samples from the animal hosts caught from three different localities. However, its DNA was detected in two pools of mesostigmatid mites, targeting the *ompB* gene, resulting in an overall positivity rate of 3.33% (Table 2). The PCR products that amplified *ompB* products are shown in Supplementary Materials Figure S1, respectively. There was no amplification of PCR products that target the *gltA* gene. Both *Rickettsia*-infected mite pools (*Laelaps* sp.) were found to parasitize *M. whiteheadi* and *B. indica*, collected from SK.

### 3.4. Phylogeny of Rickettsia

**Figure 3:**
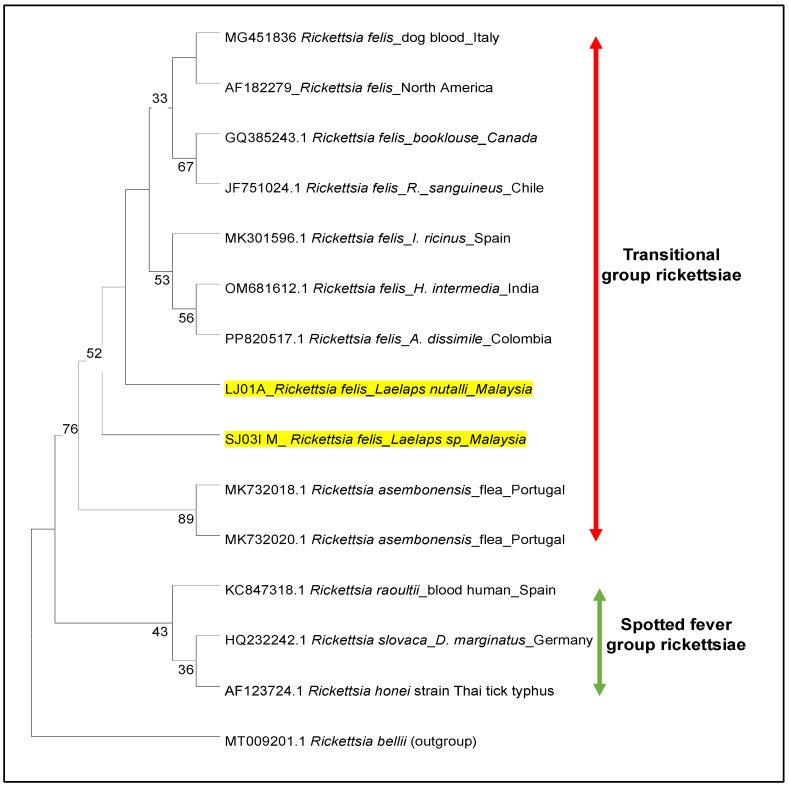
Phylogenetic tree of *Rickettsia* based on *ompB* gene sequences. The dendrogram was constructed using the neighbour-joining method with 1000 replicates in the bootstrap values. The highlight in yellow denotes the rickettsial DNA amplified from *Laelaps* spp. investigated in this study.

In this study, two positive *Rickettsia* sequences targeting the *ompB* gene revealed 100% similarity with *Rickettsia felis* sequences (Accession no. OM681612) deposited in GenBank (Figure 3). Herein, we confirmed that this study provides the first molecular evidence for *R. felis* being detected in *Laelaps* spp. in Malaysia. Based on clustering analysis of the *ompB* gene, it was revealed that the *Rickettsia* sequence from both pools of *Laelaps* sp. (LJ01A and SJ03I) was clustered in the transitional group (TG) of rickettsiae and were closely related to *R. felis* derived from several tick species and the dog blood samples (Figure 3). Moreover, the obtained sequences were separated into distinct clades from a sister clade of other TG species, *R. asembonensis*, with a 89% bootstrap value. These findings also strongly support the distinction between rickettsial species in the TG and SFG groups.

## 4. Discussion

In the present study, three localities in Selangor were chosen to determine the presence of *Rickettsia* spp. in small mammals and their on-host mesostigmatid mites. A total of 41 small mammals were collected, with agricultural land (BR) having the highest number of captured animals compared with the coastal area (BL) and recreational area (SK). The abundance and species diversity of small mammals in particular habitats are influenced by several factors, such as land use, habitat suitability, and seasonality [29]. The higher number of animals collected in the agricultural land is probably due to favourable habitats that provide better safety, moist environments, and food availability to support their development and survival [30]. Furthermore, the species of small mammals found in agricultural land indicate the role of animals as pests and opportunistic species that are less sensitive to human activities [31]. Studying the distribution and abundance of small mammals in a particular habitat is vital for identifying the potential of rodent-borne diseases in humans.

Previous studies have shown that small mammals can serve as amplifying hosts for pathogens, such as *Rickettsia* species, which are indirectly transmitted to humans through ectoparasites such as ticks, mesostigmatid mites, and chiggers [32,33,34]. This study revealed the highest infestation rate of mesostigmatid mites on small mammals caught in the recreational area, followed by coastal areas and agricultural land. Interestingly, although only a small number of animals (n = 6) were collected in SK, five of them were infested with mesostigmatid mites. SK is a recreational area that experiences human disturbance and is designated for outdoor activities, such as picnicking and camping. This ecosystem was characterized by a mixed dipterocarp forest, which provides essential ecological functions, such as carbon sequestration, along with a waterfall and serves as a habitat for wildlife. A previous study [35] investigated ectoparasite loads in small mammals across various land uses and revealed that the average ectoparasite load per individual was highest in the pastoral land, intermediate in the agricultural land and lowest in the protected parks. Their findings indicate that human disturbance increases the ectoparasite load, which aligns with the result in the present study. In addition, this study found that all individuals of *M. whiteheadi* and *B. indica* were infested with mites, indicating a preference for these rodent species by on-host mites. Previous studies have shown that *M. whiteheadi* and *B. indica* have the highest ectoparasite load, particularly with mites [36,37]. Moreover, the relatively large body size of *B. indica* contributes to its higher ectoparasite load, which is recognized as one of the largest rodent pests in Malaysia [31,38]. In contrast, *R. tiomanicus* and *R. rattus* presented low mite infestations, and none of the *T. glis* collected were infested with mites. This finding is consistent with previous studies showing that members of the family Tupaiidae have a lower rate of ectoparasite infestation than Muridae probably due to their physical fur, which is unfavourable for ectoparasites, as well as their behaviour, such as the irregular use of the nest [39,40].

In the present study, all the small mammal samples comprising blood, spleen, liver, and kidney were found to be negative for *Rickettsia* spp. Similarly, *Rickettsia* spp. were not detected in the spleens of rodents or tree shrews captured in oil palm plantations in Peninsular Malaysia [41]. In addition, the isolation and PCR detection of rickettsiae from clinical and rodent samples from areas with high antibody prevalence of human rickettsial infection in Malaysia also reported negative results [42]. In contrast, a serosurvey of wild rodents (*R. norvegicus*, *R.rattus*, *R. exulans*, *R.tiomanicus*) for rickettsioses was conducted in Thailand and Indonesia, with prevalence rates of 23.7% and 53.8%, respectively [43,44]. There was also a reported first occurrence of *R. felis* DNA in rodents in Thailand [45]. Moreover, a previous study in Lithuania successfully reported the detection of *Rickettsia* spp. in the spleens of *Micromys minutus*, *Apodemus flavicollis*, and *Myodes glareolus* [46]. Despite limited studies on the screening of *Rickettsia* spp. in Malaysia, positive detections of this pathogen have been reported in tissues (spleen, liver, and heart) of *Rattus diardii* and *Rattus norvegicus*, with a total prevalence of 13.7% [47]. Therefore, further surveillance of animal hosts should be carried out across diverse ecological regions throughout Malaysia to improve the understanding of *Rickettsia* spp. prevalence and distribution.

In this study, about 3.33% of *Laelaps* spp. were positive for rickettsiae based on the amplification of the *ompB* gene, and no PCR product was amplified using the *gltA* gene. This result suggests potential genetic variations in the *gltA* region, with which the primer used in this experiment may not be compatible. Further investigation is required to determine whether the absence of *gltA* amplification is due to primer mismatches, low bacterial load, or degradation of the target sequence.

In comparison, the role of mesostigmatid mites as vectors for rickettsiae in Malaysia remains understudied, especially when compared to other vectors such as ticks and fleas. To date, only one study has attempted to detect *Rickettsia* spp. in *Laelaps* mites, but no *Rickettsia* spp. were detected [47]. While most previous studies in Southeast Asia detected *Rickettsia* spp. in other ectoparasites, such as ticks and fleas, no *Rickettsia* spp. were detected in the *Laelaps* mite [48,49,50]. A lower positivity rate observed in this study was expected due to the small sample size of *Laelaps* sp. (n = 60) used in the screening. Similarly, a study from Taiwan, which screened *Rickettsia* spp. in a sample size of 72, reported comparable positivity rates, with 4.2% and 9.7% detected based on the *ompB* and *gltA* genes, respectively [17]. Furthermore, fewer small animals were collected at each location, which may be due to the short sampling duration (four consecutive days). It is expected that a longer sampling duration would increase the likelihood of disease detection [8]. Selangor is located on the west coast of Malaysia and experiences wet (October to April) and dry (May to September) seasons throughout the year. The study was conducted during the wet season, which may have contributed to the lower number of animals actively foraging for food. The previous study on the abundance of small mammals and their ectoparasites in Peninsular Malaysia showed that more animals were trapped during the dry season than in the wet season [51].

Sequence analysis of the partial *ompB* amplicons of both positive samples from Laelaps mites revealed 100% similarity with *R. felis.* This pathogen is an obligate intracellular bacterium that infects diverse arthropods, such as ticks and fleas, but is principally associated with cat fleas (*Ctenocephalides felis*) and is widely regarded as an emerging human pathogen [10]. To the best of our knowledge, this study is the first to report *R. felis*-positive feeding *Laelaps* spp. in Malaysia, as well as in Southeast Asia. Given the significant findings of *R. felis* in recreational areas, further sampling with expanded localities is vital to fully understand the circulation of this laelapid-associated bacterium. While *Rickettsia felis* is typically associated with fleas, the finding of the bacteria in *Laelaps* mites could indicate that these mites might also play a role in the transmission of rickettsioses, either as direct vectors or as a part of the wider ecological network of vector-borne diseases. While *Laelaps* mites are primarily ectoparasites of small mammals, human exposure could occur through direct contact with infected animals or indirectly via contaminated environments. If *Rickettsia felis* can be transmitted by these mites, it could represent a new route of infection for humans, especially in areas where mite populations are dense or widespread.

This study has several limitations that offer the potential for improvement in future investigations. First, it is recommended that future studies be conducted in areas with positive cases of rickettsiosis. For example, studies conducted in localities with known cases of rickettsial diseases and high seroprevalence showed the presence of pathogens in both vectors and small mammals [13,28]. Therefore, the transmission cycle of *Rickettsia* spp. may not be present in some sampling areas (BR and BL) of our study. Second, the sample size used in this study was relatively small compared with those used in other studies, suggesting that increasing both the sample size and the sampling duration would increase the likelihood of disease detection. Moreover, the abundance of ectoparasites can be affected by several factors, such as climate, habitat type, and the presence of the host species. Future studies may include these factors to fully understand the parameters affecting the population and mite distributions in certain areas. Finally, in this study, two target genes were used for the detection of *Rickettsia* spp., with only *ompB* showing *Rickettsia*-positivity in mesostigmatid mites. Thus, the application of PCR targeting multiple genes for pathogen detection, instead of relying on only two genes, may potentially increase the detection rate. Advanced PCR techniques, such as multiplex real-time PCR, can detect both qualitative and quantitative pathogens, as well as be capable of the simultaneous detection of several genes within a single sample.

## 5. Conclusions

To our knowledge, the present study is the first report of the presence of *R. felis* in *Laelaps* spp. from Malaysia. Although the molecular evidence showed a low infection rate of *Rickettsia* spp. in mesostigmatid mites in three localities in Selangor, further research is warranted to assess the public health implications and clarify the current status of rickettsioses in Malaysia. The circulation of rickettsiae in the ectoparasite populations of small animals in recreational areas suggests a potential health risk to the public. Furthermore, this study highlights the need for continuous surveillance of small mammal populations and their ectoparasites in various ecological areas in Malaysia. While most reported studies have focused on fleas and ticks as vectors for rickettsioses, further research should examine mite species, such as *Laelaps* mites, which are crucial for identifying novel vectors, understanding transmission cycles, and ultimately improving disease prevention in Malaysia.

## Figures and Tables

**Table 1:** Mite species were collected from small animals in Sungai Kedondong, Beranang, and Bagan Lalang.

Locality	Host Species (n Examined)	Mite Species	No. of Hosts Infested (%)	Total No. of Individual Mites	Infestation Rate (%)	Mean Intensity
Sungai Kedondong (SK)	*Maxomys whiteheadi* (3)	*Laelaps* spp.	3 (7.32)	126	7.32	42
	*Bandicota indica* (2)	*Laelaps* spp.	2 (4.88)	201	4.88	100.5
	*Rattus tiomanicus* (1)	-	0 (0)	0	0	
Total	6		5 (12.20)	327	12.20	
Beranang (BR)	*Rattus tiomanicus* (13)	*Laelaps* spp.	2 (4.88)	26	4.88	13
	*Rattus rattus* (1)	-	0 (0)			
	*Tupaia glis* (7)	-	0 (0)			
Total	21		2 (4.88)	26	4.88	
Bagan Lalang (BL)	*Rattus tiomanicus* (1)		0 (0)	0	0	
	*Rattus rattus* (7)	*Laelaps* spp.	2 (4.88)	10	4.88	5
	*Tupaia glis* (6)	-	0 (0)	0	0	
Total	14		2 (4.88)	10	4.88	
**Total location**	**41**		**9 (21.95)**	**363**	**21.96**	

Mean Intensity = the mean number of mites found on infested hosts.

**Table 2:** Positivity rates of *Rickettsia* spp. in small mammals and mesostigmatid mites using *gltA* and *ompB* targeted genes.

Sample Type	No. of Samples/Pools Examined	Target Gene		No. of Positives	Positivity Rate (%)
		*gltA*	*ompB*		
Blood	36	0	0	0	0 (0/36)
Spleen	41	0	0	0	0 (0/41)
Kidney	41	0	0	0	0 (0/41)
Liver	41	0	0	0	0 (0/41)
*Laelaps* spp.	60	0	2	2	3.33 (2/60)

## Data Availability

No new data were created or analysed in this study. Data sharing is not applicable to this article.

## Supplementary Materials

The following supporting information can be downloaded at: https://www.mdpi.com/article/10.3390/vetsci12050443/s1, Figure S1: Result of Fisher’s Exact Test to assess the significant association between infestation rate and localities.; Figure S2: Agarose gel of nested PCR of mesostigmatid mites using *OmpB* Gene.
