# Supplementary material for: First Report on the Molecular Detection and Characterization of Rickettsia felis in Laelapidae (Acari: Mesostigmata) Mites in Malaysia
Source: Vet Sci. 2025 May 6;12(5):443. doi: 10.3390/vetsci12050443 (PMC12115656; doi:10.3390/vetsci12050443)
Supplement: Supplementary file 1 [file vetsci-12-00443-s001.zip › vetsci-3523657-supplementary.pdf]

Case Processing Summary

	Cases					
	Valid		Missing		Total	
	N	Percent	N	Percent	N	Percent
Locality * Cases	41	100.0%	0	0.0%	41	100.0%

Locality * Cases Crosstabulation

Count

		Cases		Total
		Infective	Non-infective	
Locality	Bagan Lalang	2	12	14
	Beranang	2	19	21
	Sg Kedondong	5	1	6
Total		9	32	41

Chi-Square Tests

	Value	df	Asymptotic Significance (2-sided)	Exact Sig. (2-sided)
Pearson Chi-Square	15.568[a]	2	.000	.001
Likelihood Ratio	13.057	2	.001	.002
Fisher's Exact Test	12.140			.002
N of Valid Cases	41			

a. 4 cells (66.7%) have expected count less than 5. The minimum expected count is 1.32.

Figure S1. Result of Fisher's Exact Test to assess the significant association between infestation rate and localities.

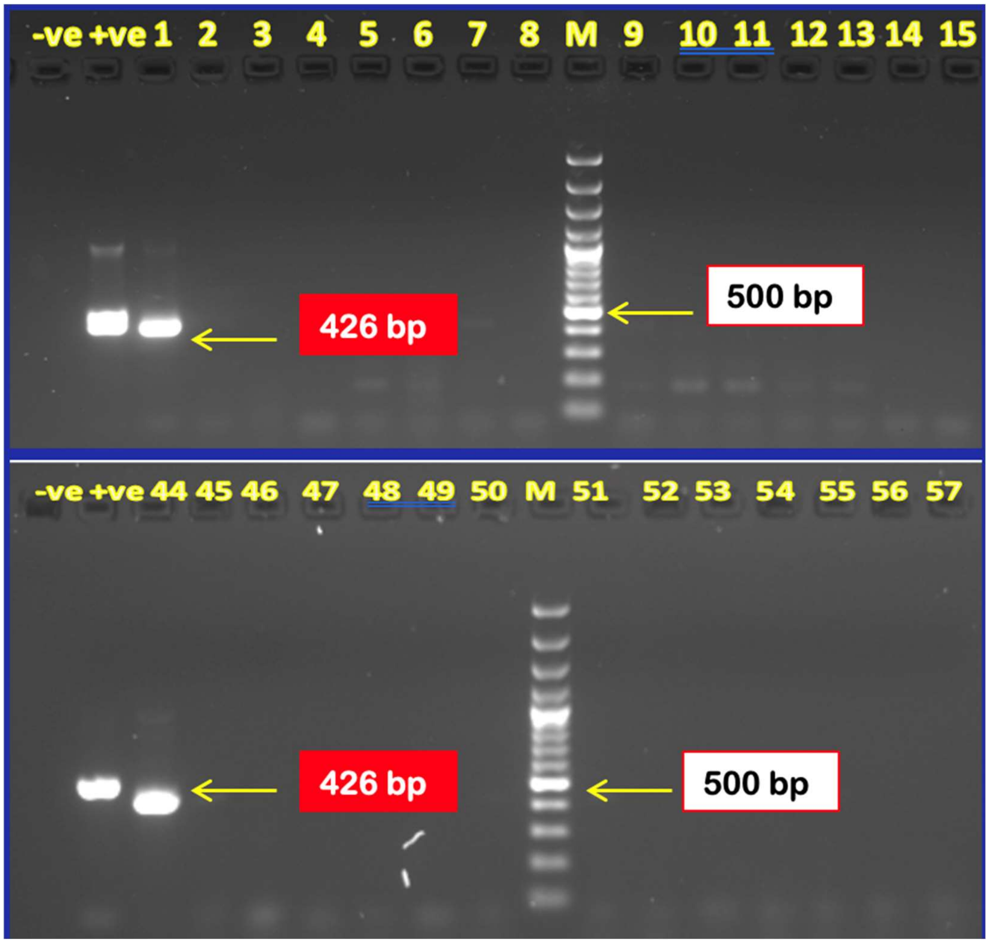

Figure S2. Agarose gel of nested PCR of mesostigmatid mites using *OmpB* Gene. Lane -ve: negative control, +ve: positive control, M: 100 bp DNA Marker, 1-15, 44-57 : DNA extract of pooled mesostigmatid mites.
